# A Wonderful Penman

**Published:** 1875-12

**Authors:** 


### A WONDERFUL PENMAN.

Mr. I. S. Preston, of 457 Herkimer St. Brooklyn, one of the subscriber's to HALL'S JOURNAL OF HEALTH, sends us some very remarkable specimens of his art as a penman. He not only *writes* wonderfully, but handles a pen much as the skilled artist wields his brush. His card states that he sends 50 elegantly written cards to any address for 50 cents, and that he fills orders for wedding-cards, monograms and all kinds of ornamental work. He is the teacher of penmanship and drawing in one of the great Seminries of learning in Brooklyn. All orders for cards are filled at the shortest notice, by this gentleman.

